# Racial Disparities in Survival Outcomes of Colorectal Cancer Patients After Surgical Resection

**DOI:** 10.7759/cureus.22064

**Published:** 2022-02-09

**Authors:** Dana Shively, Sarah S Makhani, Antoun Bouz, Elizabeth Hernandez, Katherine Chung-Bridges

**Affiliations:** 1 Medical School, Florida International University (FIU) Herbert Wertheim College of Medicine, Miami, USA; 2 Medical and Population Health Sciences Research, Florida International University (FIU) Herbert Wertheim College of Medicine, Miami, USA; 3 Translational Medicine, Florida International University (FIU) Herbert Wertheim College of Medicine, Miami, USA; 4 Public Health, Florida International University (FIU) Herbert Wertheim College of Medicine, Miami, USA; 5 Research, Health Choice Network, Miami, USA

**Keywords:** retrospective research, surgical resection, survival outcomes, racial and ethnic disparities, colorectal cancer

## Abstract

Introduction: Colorectal cancer is one of the most common cancers in the United States. Significant disparities exist among racial and ethnic minorities diagnosed with colorectal cancer compared to non-Hispanic Whites. However, understanding of survival outcomes following curative surgical resection in this population is limited.

Objective: To evaluate the association between race and colorectal cancer-specific mortality in patients who were treated with major surgical resection of the colon.

Materials and Methods: This study was a retrospective cohort analysis using the Surveillance, Epidemiology, and End Results (SEER) Program database from 2010 to 2016. The patient population consisted of adult patients (≥18 years old) diagnosed with a primary malignancy of colorectal cancer treated with major surgical resection of the colon. The main outcome measures were survival time at one and five years following diagnosis and cancer-specific death.

Results: A total of 120,598 patients with primary colorectal malignancy treated with surgical resection of the colon were identified. Across all racial groups, most patients presented with moderately differentiated colorectal cancer. Non-Hispanic Blacks had the highest proportion of diffuse metastases (p<0.001). After adjusting for covariates, Hispanic respondents had the lowest one-year survival (adjusted HR: 1.26, 95%CI (1.21-1.31) and five-year survival when compared to Whites (adjusted HR: 1.13, 95%CI: 1.10-1.15). Factors associated with a shorter survival include age ≥ 70 years old, unmarried status, metastatic disease, and high-grade tumors (p<0.001).

Conclusions: Racial disparities exist in the overall survival of patients with colorectal cancer who are treated with surgical resection of the colon. Hispanic patients had the highest hazard of death, followed by Non-Hispanic Asian-Pacific Islanders and Non-Hispanic Blacks, compared to Whites. While surgical resection can be curative, the quality and accessibility of post-operative care may differentiate survival outcomes among racial groups.

## Introduction

Worldwide, colorectal cancer (CRC) accounts for nearly 10% of all annually diagnosed cancers and cancer-specific deaths [1]. Current projections estimate that the incidence of CRC worldwide will increase to 2.5 million new cases in 2035 [2]. It is the third most common cancer among men and women in the United States (US), with significant disparities between racial and ethnic minorities compared to Non-Hispanic Whites (NHW) [3]. According to the American Cancer Society, minorities are more likely to be diagnosed with cancer and have higher mortality rates compared to the general United States population. This disparity is particularly significant in CRC. Asian/Pacific Islander (Asian/PI) individuals are at the lowest risk for developing colorectal cancer. The incidence of colorectal cancer is highest in Non-Hispanic Blacks (NHB), with incidence rates approximately 20% greater than NHW and 50% greater than Asian/PIs. [4]

An even more stark disparity is appreciable in death rates, as CRC-specific mortality in NHB is 19 per 100,000 population, which is twice that of Asian/PIs (9.5 per 100,000), and 40% greater than NHW (13.8 per 100,000) [5]. Despite the perceived improvements and availability in screening and treatment options for CRC, the disparity for NHB compared to NHW has been corroborated in other studies [3,6-8]. This gap is largely attributable to differences in access to healthcare and group-specific risk factor prevalence, which are intimately tied to social determinants of health and socioeconomic status. Thus, identifying key social determinants of health will play a vital role in improving the outcomes for at-risk populations.

Currently, surgical resection remains the standard of care for both staging and treating non-metastatic colon cancer. Significant advancements over the recent years have led to better surgical management options and a range of techniques for patients undergoing major resection of the colon [9]. The implementation of nationwide CRC screening is predicted to subsequently result in an increase in patients needing curative treatment with major surgical resection [6]. Although certain racial disparities in overall CRC mortality have been established, there is limited data on the role that these socioeconomic factors play in the survival outcomes of patients specifically after undergoing curative surgical resection. The aim of this study was to evaluate the association between race and CRC-specific survival among patients undergoing major resection of the colon in the US during 2010-2016.

## Materials and methods

Study design

This retrospective cohort study used secondary data analysis from the Surveillance, Epidemiology, and End Results (SEER) Program from 2010 to 2016. The database collects and publishes cancer incidence and survival data from population-based cancer registries that cover approximately 34% of the US population. Data was extracted using SEER*Stat software.

Study population and measures

This study included adult participants (≥18 years old) diagnosed with a primary malignancy of colorectal cancer as defined by the International Classification of Diseases (ICD-O-3) codes for colon and rectal cancers (C180, C182, C183, C184, C185, C186, C187, C188, C189, C199, and C209) between 2010 and 2016 in the US. Additionally, inclusion criteria included respondents undergoing major resection of the colon including partial colectomy, subtotal colectomy/hemicolectomy, total colectomy, total proctocolectomy, and colectomy with resection of contiguous organs, defined by SEER Surgery of Primary Site Coding. Patients diagnosed at autopsy or death certificate were excluded from the study, as well as respondents with missing information on survival and race/ethnicity data.

The main outcome of this study was survival time at one and five years following diagnosis of colorectal cancer. The primary endpoint was cancer-specific death, calculated from the time of diagnosis until death due to CRC. Demographic information included age at diagnosis (categorized into ages≥18-49, 50-59, 60-69, and 70+), sex (male or female), insurance status (private, Medicaid, uninsured, and insurance not specified), and marital status (married or non-married). Marital status was defined using SEER variables “married” and combining “single (never married)”, “separated”, “divorced”, “widowed”, or “unmarried partner” as unmarried. The tumor stage was categorized as distant, localized, and regional. Tumor grade, defined by ICD-O-2 coding, was divided into well-differentiated, moderately differentiated, poorly differentiated, and undifferentiated. Site of CRC was also included, categorized by proximal colon, distal colon, and rectal. Metastases to bone, liver, and lung were also studied.

Statistical analysis

Data analysis was conducted using Stata/MP version 15.2 (StataCorp LLC, College Station, Texas). Baseline characteristics were reported for demographic and socioeconomic variables, including percentages for nominal and categorical variables. Following the descriptive statistics, a bivariate chi-squared analysis was conducted to identify possible confounders. Log-rank and Kaplan Meier (KM) curves were used to compare survival between the racial groups. Pearson’s correlation coefficient analysis was conducted to determine collinearity. Unadjusted and adjusted Cox regression models were used to calculate hazard ratios (HR) and the corresponding 95% confidence intervals (CI). 

## Results

Between 2010 and 2016, there were 120,598 patients with a primary malignancy of CRC who underwent surgical resection in the US. Baseline characteristics of patients with colorectal cancer at diagnosis according to race and ethnicity are presented in Table 1. NHB respondents had a higher proportion of non-married participants (59.2%) compared to NHW (40.2%). Further, NHW respondents had a higher proportion of privately insured individuals (74.0%) compared to NHB (58.3%). Among all participants (n=120,598) in the present study, there was a higher frequency of respondents that identified as White (68.3%). Those that identified as Black had a higher proportion of participants that had moderately differentiated disease (75.0%) and a higher proportion of the population between the ages of 60-69 years (29.0%); whereas those participants who identified as NHW had a similar proportion of moderately differentiated disease (72.8%), but had a greater proportion of the population over 70 years of age (39.8%). Of those of Hispanic ethnicity, a higher percentage presented with moderately differentiated grade CRC (73%) compared to poorly differentiated (11.4%) and undifferentiated (2.2%). Further, they were more likely to be regionally staged (49.4%) rather than distant (9.1%). The highest proportion of well-differentiated CRC was found among people identifying as American Indian (12.6%), when compared to all other racial and ethnic groups (NHW, 10.6%; NHB, 11.5%; Asian/PI, 9.0%; Hispanic, 12.2%). Among all groups, the Asian/PI respondents had a higher frequency of individuals that underwent a partial colectomy (60.0%) vs. NHB (41.7%). The highest proportion of married participants was Asian/PIs (67.7%) compared to the other race and ethnicity groups.

**Table 1:** Baseline characteristics of patients with CRC post-surgical resection by racial group. CRC: colorectal cancer; PI: Pacific Islander

		Race/Ethnicity											
Characteristics		White		Black		Asian/ PI		American Indian		Hispanic		p-value	
		No.	%	No.	%	No.	%	No.	%	No.	%		
Survival (years)^1^		3.9	68.3	3.5	10.8	3.6	8.7	3.5	0.7	3.3	11.4	<0.001	
Age (years)												<0.001	
18-49		9,864	12.2	2,092	15.7	1,560	14.4	148	16.4	2,948	20.3		
50-59		17,436	21.5	3,748	28.1	2,567	23.7	249	27.6	3,944	27.2		
60-69		21,510	26.5	3,875	29	3,018	27.9	247	27.3	3,763	25.9		
70+		32,209	39.8	3,622	27.2	3,679	34	259	28.7	3,860	26.6		
Sex												<0.001	
Male		42,138	52	6,383	47.9	5,684	52.5	456	50.5	7,583	52.2		
Female		38,881	48	6,954	52.1	5,140	47.5	447	49.5	6,932	47.8		
Insurance												<0.001	
Private		58,870	74	7,604	58.3	6,704	63	414	47.3	8,350	58.5		
Medicaid		6,604	8.3	2,447	18.7	2,183	20.5	333	38.1	3,365	23.6		
Uninsured		1,770	2.2	786	6	265	2.4	7	0.8	725	5.1		
Unknown I		12,403	15.5	2,207	17	1,502	14.1	121	13.8	1,827	12.8		
Marital Status												<0.001	
Married		46,029	59.8	5,083	40.8	6,947	67.7	413	53.8	7,966	57.9		
Not Married		31,008	40.2	7,390	59.2	3,320	32.3	355	46.2	5,784	42.1		
Grade												<0.001	
Well- differentiated		8,050	10.6	1,430	11.5	922	9	105	12.6	1,667	12.2		
Moderately differentiated		55,469	72.8	9,352	75	7,848	76.7	635	76.1	10,010	73		
Poorly differentiated		10,311	13.5	1,423	11.4	1,277	12.5	71	8.5	1,724	12.6		
Undifferentiated		2,331	3.1	269	2.2	186	1.8	24	2.9	306	2.2		
Summary Stage												<0.001	
Localized		37,071	46.1	5,878	44.3	4,513	42.1	384	43	6,027	41.9		
Regional		37,132	46.1	6,185	46.6	5,314	49.5	430	48.2	7,100	49.4		
Distant		6,313	7.8	1,200	9.1	901	8.4	79	8.8	1,255	8.7		
Metastasis to Bone												0.679	
Yes		88	0.1	20	0.2	12	0.1	1	0.1	20	0.1		
No		80,451	99.9	13,244	99.8	10,674	99.9	896	99.9	14,356	99.9		
Metastasis to Liver												0.006	
Yes		3,557	4.4	679	5.1	496	4.6	42	4.7	677	4.7		
No		77,009	95.6	12,583	94.9	10,198	95.4	856	95.3	13,714	95.3		
Metastasis to Lung												<0.001	
Yes		713	0.9	163	1.2	121	1.1	9	1	156	1.1		
No		79,803	99.1	13,102	98.8	10,578	98.9	890	99	14,217	98.9		
Type of Surgery												<0.001	
Partial colectomy		39,903	49.2	5,563	41.7	6,490	60	446	49.4	7,696	53		
Subtotal colectomy		35,506	43.8	6,975	52.3	3,678	34	389	43.1	5,755	39.7		
Total Colectomy		4,089	5.1	547	4.1	462	4.2	53	5.9	736	5		
Total Proctocolectomy		557	0.7	79	0.6	53	0.5	1	0.1	100	0.7		
Colectomy with Resection		964	1.2	173	1.3	141	1.3	14	1.5	228	1.6		
CRC Subsite												<0.001	
Proximal Colon		38,472	47.5	6,976	52.3	3,691	34.1	387	42.9	6,060	41.8		
Distal Colon		22,135	27.3	4,003	30	3,975	36.7	274	30.3	4,520	31.1		
Rectal		20,412	25.2	2,358	17.7	3,158	29.2	242	26.8	3,935	27.1		
^1^Median survival time													

**Figure 1:**
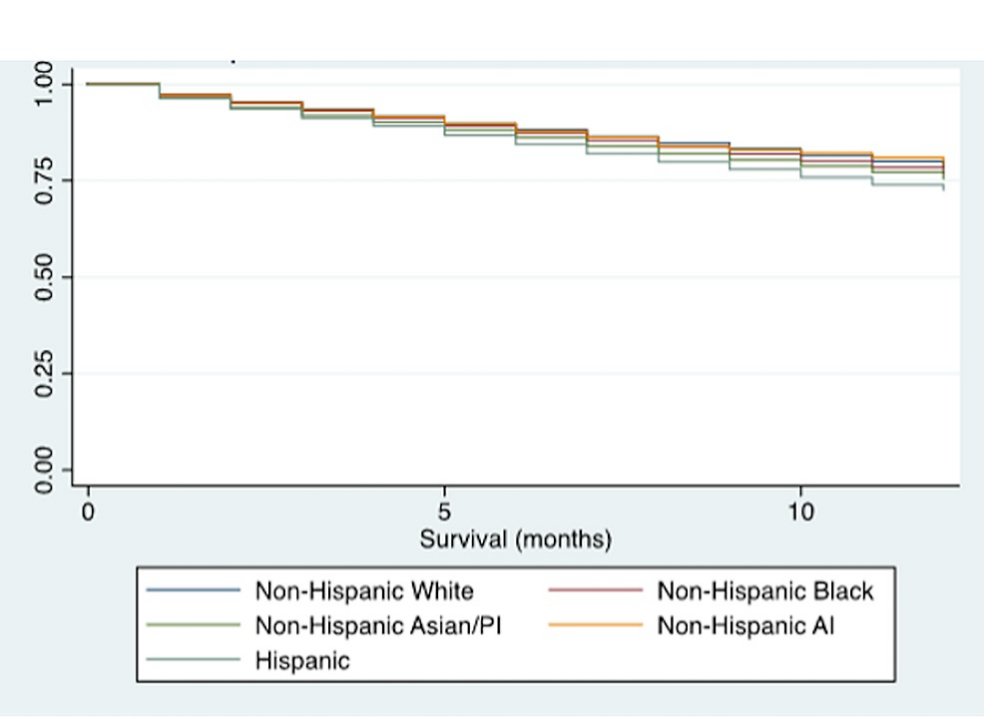
Kaplan-Meier one-year survival estimates

**Figure 2:**
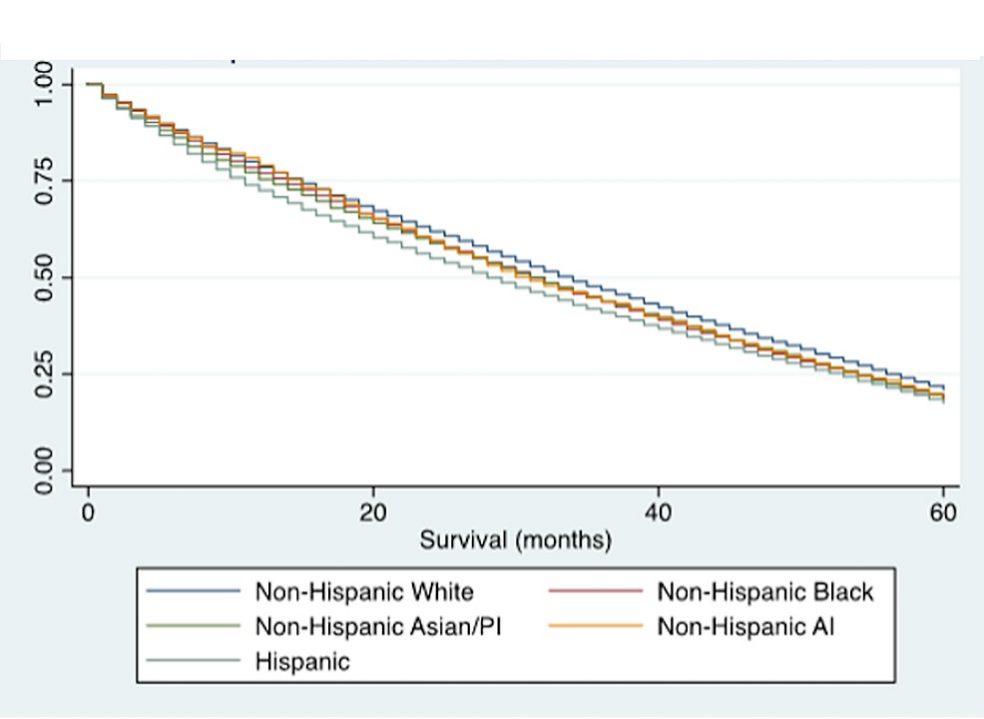
Kaplan-Meier five-year survival estimates

Unadjusted and adjusted Cox regression for one and five year survival in varying baseline characteristics using univariate and multivariate models are presented in Table 2. The proportional hazard assumptions in the Cox regression model were evaluated by comparing them to Kaplan Meier curves for survival. The observed KM expected curves were similar to the Cox predicted curves, demonstrating that the proportional hazards assumption has not been violated. After adjustment for covariates (age, sex, marital status, and area of residence), Hispanic patients had the shortest survival time, when compared to Whites (adjusted (adj) HR: 1.13, 95%CI: 1.10-1.15). Log rank tests and Kaplan Meier curves were calculated to compare survival according to race with Log rank x^2^=299.2 (p<0.001) for one-year survival, Log rank x^2^=263.7 (p<0.001) for five-year survival, and P<0.001 reflected in the Kaplan Meier curves (Figure 1 and Figure 2). Further, NHB had a higher hazard of mortality at five-years, when compared to White patients (adj HR: 1.04, 95%CI: 1.02-1.07). Respondents receiving Medicaid had a 22% increased hazard of mortality, when compared to those with private insurance (95%CI: 1.19-1.25). As expected, patients who were over 70 years old, unmarried, had metastases, or had high-grade tumors had a lower survival outcome than their counterparts (p<0.001).

**Table 2:** Unadjusted and adjusted Cox regression for one- and five-year survival. HR: hazard ratio; CI: confidence interval; Ref: reference category; PI: Pacific Islander; NOS: insurance not otherwise specified

	One Year Survival				Five Year Survival			
Characteristics	Unadjusted		Adjusted^a^		Unadjusted		Adjusted	
	HR	(95%CI)	HR	(95%CI)	HR	(95%CI)	HR	(95%CI)
Race/Ethnicity								
White	Ref		Ref		Ref		Ref	
Black	1.08	(1.04-1.12)	1.04	(1.00-1.09)	1.08	(1.06-1.10)	1.04	(1.02-1.07)
Asian/PI	1.17	(1.12-1.22)	1.14	(1.10-1.20)	1.08	(1.06-1.11)	1.07	(1.04-1.10)
American Indian	0.98	(0.85-1.13)	0.92	(0.78-1.08)	1.06	(0.99-1.14)	0.98	(0.90-1.06)
Hispanic	1.33	(1.29-1.38)	1.26	(1.21-1.31)	1.16	(1.13-1.18)	1.13	(1.10-1.15)
Age (years)								
18-49	Ref		Ref		Ref		Ref	
50-59	0.97	(0.93-1.01)	1.01	(0.97-1.05)	0.98	(0.96-1.00)	1	(0.98-1.02)
60-69	0.97	(0.93-1.01)	1.02	(0.98-1.06)	0.89	(0.96-1.00)	1.01	(0.98-1.03)
70+	1.12	(1.08-1.16)	1.2	(1.15-1.26)	1.06	(1.04-1.08)	1.1	(1.08-1.12)
Sex								
Female	Ref		Ref		Ref		Ref	
Male	0.99	(0.96-1.01)	1.03	(1.00-1.05)	1.01	(1.00-1.03)	1.05	(1.03-1.06)
Insurance								
Private	Ref		Ref		Ref		Ref	
Medicaid	1.43	(1.38-1.48)	1.34	(1.29-1.39)	1.28	(1.25-1.31)	1.22	(1.19-1.25)
Uninsured	1.03	(0.96-1.10)	1	(0.92-1.08))	0.99	(0.96-1.03)	0.96	(0.92-1.00)
Insurance NOS	1.05	(1.01-1.08)	1	(0.97-1.04))	1.04	(1.02-1.06)	1.02	(1.00-1.04)
Marital Status								
Married	Ref		Ref		Ref		Ref	
Not Married	1.19	(1.16-1.22)	1.13	(1.10-1.16)	1.12	(1.11-1.13)	1.09	(1.08-1.11)
Grade								
Well-differentiated	Ref		Ref		Ref		Ref	
Moderately differentiated	0.97	(0.93-1.01)	0.94	(0.90-0.98)	0.99	(0.96-1.01)	0.97	(0.95-0.99)
Poorly differentiated	1.07	(1.02-1.12)	0.97	(0.92-1.02)	1.01	(0.98-1.03)	0.96	(0.93-0.99)
Un-differentiated	1.35	(1.25-1.45)	1.23	(1.14-1.33)	1.2	(1.15-1.25)	1.15	(1.10-1.20)
Stage								
Distant	Ref		Ref		Ref		Ref	
Localized	0.55	(0.53-0.57)	0.69	(0.64-0.74)	0.63	(0.62-0.65)	0.74	(0.71-0.77)
Regional	0.68	(0.65-0.71)	0.86	(0.81-0.92)	0.7	(0.68-0.72)	0.82	(0.79-0.85)
Metastasis to Bone								
No	Ref		Ref		Ref		Ref	
Yes	2.33	(1.83-2.98)	1.44	(1.09-1.90)	2.03	(1.72-2.42)	1.24	(1.02-1.50)
Metastasis to Liver								
No	Ref		Ref		Ref		Ref	
Yes	1.79	(1.71-1.87)	1.38	(1.28-1.49)	1.62	(1.57-1.66)	1.26	(1.20-1.32)
Metastasis to Lung								
No	Ref		Ref		Ref		Ref	
Yes	1.98	(1.81-2.17)	1.39	(1.25-1.55)	1.93	(1.81-2.04)	1.44	(1.34-1.54)
Type of Surgery								
Partial colectomy	Ref		Ref		Ref		Ref	
Subtotal colectomy	1.04	(1.02-1.06)	0.92	(0.89-0.95)	1.02	(1.01-1.04)	0.96	(0.94-0.98)
Total colectomy	0.95	(0.89-1.00)	0.96	(0.90-1.02)	1.02	(0.99-1.05)	1.01	(0.98-1.05)
Total pro- ctocolectomy	0.92	(0.79-1.07	0.97	(0.82-1.14)	0.99	(0.92-1.07)	1.03	(0.95-1.12)
Colectomy with resection	1.19	(1.08-1.32)	1.06	(0.95-1.19)	1.1	(1.04-1.16)	0.98	(0.92-1.05)
CRC Subsite								
Proximal Colon	Ref		Ref		Ref		Ref	
Distal Colon	0.95	(0.93-0.98)	0.91	(0.88-0.94)	0.96	(0.94-0.98)	0.93	(0.91-0.95)
Rectal	0.85	(0.82-0.87)	0.81	(0.78-0.84)	0.93	(0.91-0.94)	0.9	(0.88-0.91)
^a ^This model adjusted for age, sex, race, ethnicity, marital status, and area of residence								

## Discussion

This study found racial and ethnic disparities in CRC-specific mortality after surgical resection of the colon. Racial and ethnic minorities had a higher hazard of mortality over one and five years following surgery, compared to their White counterparts. Specifically, we observed that Hispanic respondents had the highest hazard of post-surgical mortality, after adjusting for age, sex, marital status, and area of residence. They were followed by Asian/PIs and NHB for an increased hazard of CRC-specific mortality following surgery.

Overall, the present study is consistent with the existing literature. Many studies have shown that racial and ethnic minorities have higher CRC-related mortality compared to Whites [4,10-12]. While Hispanic patients historically had better survival outcomes than White respondents; this has recently shifted in the literature as recent studies reveal staging and survival benefits are decreasing for Hispanic patients with colon cancer [13,14]. Stage-adjusted survival outcomes have been seen to be worse for Hispanics (specifically Mexicans) when compared with NHW patients [15]. A possible explanation for worsening survival outcomes could be the reduced up-to-date screening for Hispanics (47%) when compared to the national average (62%) [16].

This study is one of the first to assess this relationship following curative surgical resection of the colon. In this study, racial and ethnic minorities had significantly worse one- and five-year prognoses than Whites, even after surgery. One study demonstrates racial and socioeconomic disparities in surgical complications and in-hospital mortality among patients with CRC in the US [6]. The authors specifically note that among those patients who received surgery, Black patients, as well as patients with Medicaid and Medicare insurance, experienced a higher number of post-surgical complications (Black OR = 1.07 95% CI 1.01-1.13), and high in-hospital mortality (Black OR = 1.18, 95% CI 1.00-1.39) compared to White patients. There was also higher in-hospital mortality for those with Medicare (OR=1.23 95% CI 1.05-1.45) and Medicaid (OR=1.66 95% CI 1.23-2.22) compared to those patients with private insurance [6]. Others have noted a similar finding, where socioeconomic status seemed to be an important prognosticator of survival, specifically for African American patients [17]. A ten-year retrospective study reported a significant disparity in overall and disease-free survival between Black and White patients; however, the authors were unable to identify a clear, attributable cause, as there were no significant differences in the baseline presentation of each population. Both subsets of patients had similar colorectal stages at presentation and no obvious difference in curative surgical resection, adjuvant chemotherapy, and adjuvant radiotherapy. This finding highlights the importance of a detailed prospective analysis to help discover determinants and solutions for the disparities that exist and that reductions in survival for Black patients with CRC were not related to differences in socioeconomic status but may be due to a combination of biological and other non-cancer related health conditions [18]. This could also be the case for other ethnic minorities, like Hispanics and Asian/PIs.

This study revealed that CRC survival following curative surgery varied by factors other than race, including sex and insurance status. The male respondents appear to have a higher hazard of CRC-specific mortality compared to their female counterparts. Additionally, Medicaid respondents have an increased likelihood of mortality at one and five years following surgery, compared with the privately insured. This finding is consistent with the literature, which shows disparities in treatment and outcomes of CRC in patients with Medicaid compared to those who are insured [19].

As anticipated, those who are older (>70 years), have high grade tumors, and have metastases are less likely to survive than their counterparts. Unmarried patients with CRC are more likely to have decreased survival at one and five years following surgery, compared to their married counterparts. In a recent study, researchers found that unmarried patients with surgically treated colorectal cancer had a worse five-year disease-specific survival after adjusting for cofounders, with further stratified analysis revealing that unmarried status was a significant negative factor in those patients age >65, female, and well/moderately differentiated tumor [20]. The authors hypothesized that this difference is likely due to the influence a spouse has on their partner's health-related behavior, financial assistance, and emotional support. Conversely, those with CRC in the distal colon and rectum have a higher likelihood of survival than those with CRC in the proximal colon. This aligns with the literature, which suggests that differences in risk factors and tumor characteristics across sites within the colon and rectum may translate to variations in survival outcomes [21,22].

Limitations

The results of this study should be considered within the context of several limitations. There are many socioeconomic factors that may contribute to the overall survival of patients with CRC, which were not available for evaluation in this study. Indicators such as access to hospitals, patient income, and patient education levels can all contribute to the patient's prognosis. Genetic biomarkers of sporadic and hereditary hypermutated and non-hypermutated CRC, such as hMSH6, hMSH2, hMSH3, hMLH3, POLE, APC, TP53, KRAS, TTN, BRAF, and ACVR2A were not available [23]. These markers reflect earlier onset and can increase the risk of multiple cancers, thereby worsening the prognosis. Additionally, external validity through SEER is limited, as 34% of all cancers are depicted in the SEER database. Future studies should incorporate larger representation in order to strengthen external validity.

## Conclusions

In conclusion, the elements that lead to the survival of colorectal cancer are multifactorial. While some of the pathogenesis of racial disparities can be attributed to biology and genetics, socioeconomic factors play a large role in determining a patient’s prognosis. Although all patients included in the present study underwent surgical resection as a component of their treatment plan for CRC, our results demonstrate significant racial disparities contributing to the differential survival rates observed between racial groups. Compared to NWB patients, Hispanic patients had a shorter survival time, followed by Asian/PIs and NHB. While surgical resection can be curative in CRC, the quality and accessibility of post-operative care may differentiate survival outcomes among racial groups. Future studies should investigate the presence of CRC-specific genetic markers among racial groups in order to determine if genetic factors can further explain the disparity in survival.
